# Tenascin C Promiscuously Binds Growth Factors via Its Fifth Fibronectin Type III-Like Domain

**DOI:** 10.1371/journal.pone.0062076

**Published:** 2013-04-18

**Authors:** Laura De Laporte, Jeffrey J. Rice, Federico Tortelli, Jeffrey A. Hubbell

**Affiliations:** 1 Institute of Bioengineering, School of Life Sciences and School of Engineering, Ecole Polytechnique Fédérale de Lausanne, Lausanne, Switzerland; 2 Department of Chemical Engineering, Tennessee Technological University, Cookeville, Tennessee, United States of America; 3 Institute of Chemical Sciences and Engineering, School of Basic Sciences, Ecole Polytechnique Fédérale de Lausanne, Lausanne, Switzerland; University of California, San Diego, United States of America

## Abstract

Tenascin C (TNC) is an extracellular matrix protein that is upregulated during development as well as tissue remodeling. TNC is comprised of multiple independent folding domains, including 15 fibronectin type III-like (TNCIII) domains. The fifth TNCIII domain (TNCIII5) has previously been shown to bind heparin. Our group has shown that the heparin-binding fibronectin type III domains of fibronectin (FNIII), specifically FNIII12–14, possess affinity towards a large number of growth factors. Here, we show that TNCIII5 binds growth factors promiscuously and with high affinity. We produced recombinant fragments of TNC representing the first five TNCIII repeats (TNCIII1–5), as well as subdomains, including TNCIII5, to study interactions with various growth factors. Multiple growth factors of the platelet-derived growth factor (PDGF) family, the fibroblast growth factor (FGF) family, the transforming growth factor beta (TGF-β) superfamily, the insulin-like growth factor binding proteins (IGF-BPs), and neurotrophins were found to bind with high affinity to this region of TNC, specifically to TNCIII5. Surface plasmon resonance was performed to analyze the kinetics of binding of TNCIII1–5 with TGF-β1, PDGF-BB, NT-3, and FGF-2. The promiscuous yet high affinity of TNC for a wide array of growth factors, mediated mainly by TNCIII5, may play a role in multiple physiological and pathological processes involving TNC.

## Introduction

**Figure 1:**
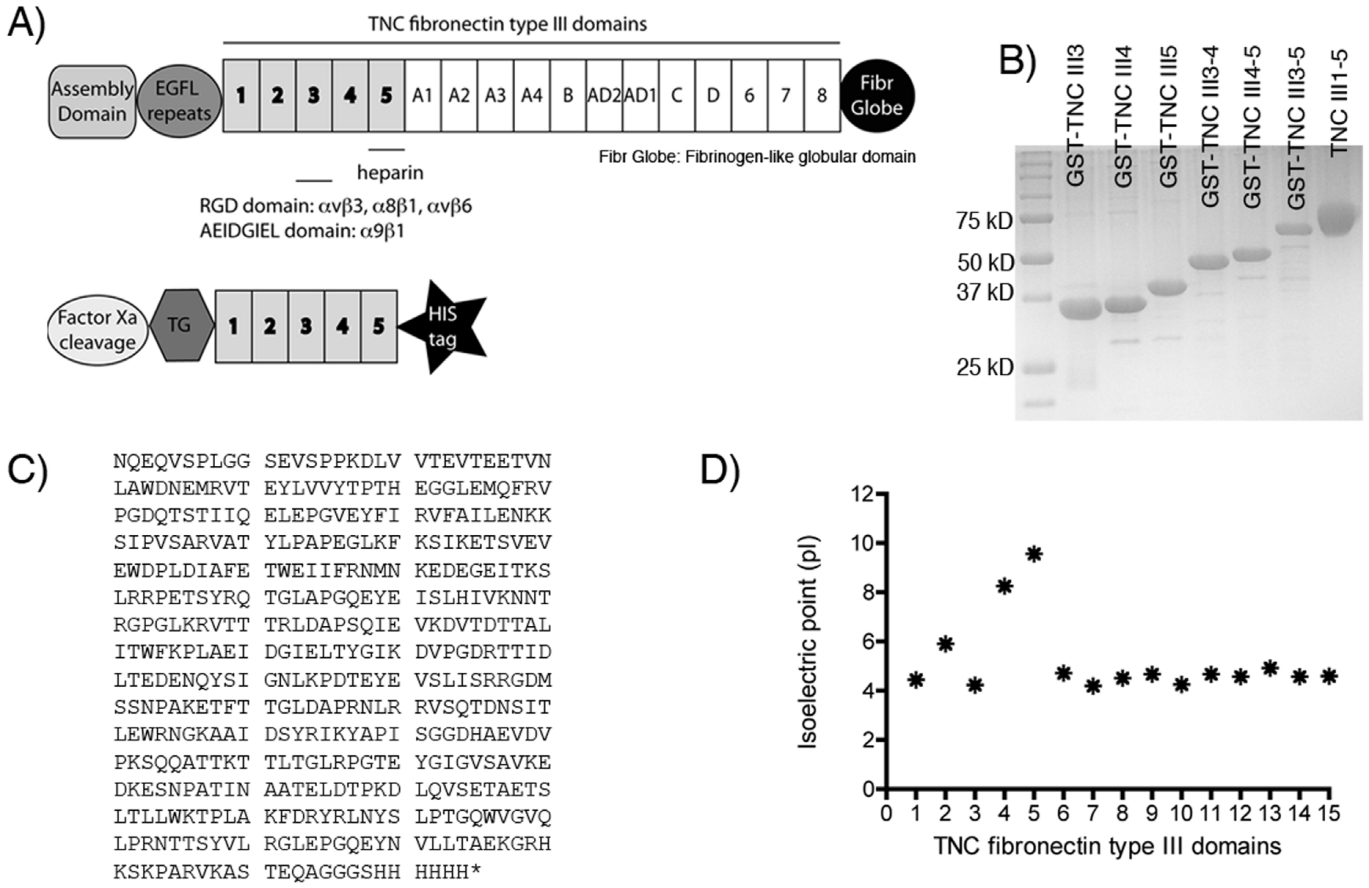
Production and characterization of TNCIII1-5. (A) Top: schematic of structural domains of full length human tenascin C (TNC), with major integrin binding and heparin binding domains. Bottom: schematic of the designed TNCIII1-5 fragment. (B) SDS-PAGE gel of purified TNCIII1–5 with the molecular weight of 53 kD, and the different GST-TNCIII (fusion) domains (GST-TNCIII3, GST-TNCIII4, GST-TNCIII5, GST-TNCIII3-4, GST-TNCIII4-5, GST-TNCIII3-5). (C) Amino acid sequence of TNCIII1–5. (D) Graphical depiction of isoelectric points of the TNCIII domains.

Tenascin C (TNC) is a complex multifunctional extracellular matrix (ECM) glycoprotein forming a disulfide-bonded hexabrachion. The monomeric unit consists of four major domains (Figure 1, A) [1], [2]. The N-terminal domain mediates hexamerization by forming a coiled structure with interchain disulfide bonds. This is followed by a series of 14.5 epidermal growth factor-like (EGFL) repeats, which are between 30 and 50 amino acids long and each contain six cysteines; these EGFL repeats have anti-adhesive properties that encourage migration. The third domain consists of a series of up to 15 fibronectin type III-like (TNCIII) repeats, which are approximately 90 amino acids long and form two sheets of antiparallel β-strands [3]. This region, which contains several integrin-binding sites that promote cell adhesion, is subject to extensive alternative splicing, generating many isoforms [3]. Finally, a fibrinogen-like globular domain is located at the C terminus.

TNC is a multifaceted protein that is expressed throughout development and is present within healthy adult tissues at low levels [4], [5], [6]. In addition, TNC is expressed in adults under tight spatio-temporal control during tissue remodeling, such as wound healing [7] and nerve regeneration [8], as well as during inflammation [2]. During development, TNC plays a highly regulated and dynamic role in the patterning of the skeletal, neural and vascular systems [3]. TNC has been shown to affect cell adhesion, proliferation, and migration [3] and to modulate stem cell behavior [1]. During physiological wound repair, TNC is produced in a tightly controlled, rapid, and transient manner. However, uncontrolled TNC production has been observed in cases of abnormal tissue growth associated with cancer [9], fibrotic diseases, chronic wounds, cardiovascular diseases [10], [11], autoimmune diseases [2], and restenosis after percutaneous coronary angioplasty [12] and stent implantation [13]. Recently, TNC has also been implicated in cardiac and arterial inflammation, tumor angiogenesis and metastasis [14], [15].

TNC has been extensively studied and found to interact directly with a variety of cell types through binding to integrins, heparan sulfate proteoglycans, and other cellular receptors as well as indirectly via binding to fibronectin [16], [17]. The location of the heparin-binding domain has been mapped to the fourth and principally fifth TNCIII domain [17], [18]. Recently, our group has demonstrated that a similar heparin-binding fibronectin type III region within fibronectin (FNIII), FNIII12–14, binds a large number of growth factors with high affinity [19].

The observation of promiscuous growth factor binding to FNIII12–14 described above motivated us to examine potential growth factor interactions with TNC, specifically within its heparin-binding region. Here, we report that the heparin-binding region of TNC has a high affinity towards a large number of growth factors from the vascular endothelial growth factor (VEGF)/platelet-derived growth factor (PDGF), fibroblast growth factor (FGF), transforming growth factor-β (TGF-β) and neurotropin families, as well as from the insulin-like growth factor-binding proteins (IGF-BPs), with nanomolar binding affinities. The finding that TNC has the ability to bind growth factors and that the fifth TNCIII domain (TNCIII5) plays a major role in its binding activity may have important implications in the functions of this dynamic ECM protein in (patho)biology.

## Methods

### Production of tenascin C fragments

Human TNCIII1–5 was expressed in mammalian cells, HEK-293E, using the vector pSecTag A (Invitrogen), which uses an Ig kappa leader sequence for secretion of the TNC fragment. A SfiI cloning site, which codes for the amino acids AAQPA, follows the signal sequence. Immediately after the cloning site, a factor Xa cleavage site was introduced to allow for complete removal of the leader peptide. A transglutaminase (TG) substrate, residues 1–8 of alpha2-plasmin inhibitor (NQEQVSPL), was designed to follow the factor Xa cleavage site and thus be present at the N-terminus of the mature, secreted protein. A short linking sequence of GGS was added before the addition of the TNCIII1–5, namely residues E622 to A1074 of TNC. At the C-terminus of the construct is a short linker sequence, GGGS, followed by a 6xHis tag (Figure 1, C).

HEK-293E cells were transfected with 1.25 µg of plasmid per 10^5^ cells per 1 mL of final growth medium (Excell 293, 4 mM glutamine, 3.75 mM valproic acid). The culture medium was harvested after 7 days of shaker flask expression and cells were removed by filtration. The protein was then purified using an FPLC (Akta Explorer, GE Healthcare) with a HisTrap HP column (GE Healthcare). After elution of the protein, the buffer was changed by dialysis in tris buffer (20 mM Tris-HCl, 150 mM NaCl, pH 7.4). The lipopolysaccharide (LPS) levels were detected with a HEK-BlueTM LPS Detection Kit (InvivoGen).

The flanking amino acids of the domains produced from TNC are shown within parenthesis. Genes for GST-TNCIII3 (T801–T891), GST-TNCIII4 (T891–T983), GST-TNCIII5 (T983–A1074), GST-TNCIII3-4 (T801–T983), GST-TNCIII4-5 (T891–A1074), and GST-TNCIII3-5 (T801–A1074) were cloned into pGEX-6p-1 using BamHI and EcoRI restriction sites and DH5alpha E. coli. Each gene sequence was verified using capillary sequencing services (Fasteris). Proteins were expressed in BL21 E. coli and purified using Ni–NTA agarose (Qiagen) according to manufacturers instructions. The proteins were dialyzed into phosphate buffered saline (PBS, Invitrogen), pH 7.4. The TNC fragments were run on a SDS-PAGE gel and stained with SimplyBlue (Invitrogen).

The protein pI of the different TNCIII domains is a theoretical calculation using the residues from each TNCIII domain and the pK values of amino acids described in Bjellqvist et al. [20]

Full-length TNC was purchased from (AdB serotech), and SDS-PAGE confirmed its purity and molecular weight.

### Detection of growth factor binding via ELISA

To detect binding of TNCIII1-5 to growth factors, growth factors of human sequence (at 50 nM in PBS) (VEGF-A165 (Invitrogen), PDGF-BB (Gibco), others (Peprotech)) were coated on an ELISA plate (1 h, 37°C). After blocking with 2% bovine serum albumin (BSA, Sigma) (1 h, RT), 100 nM TNCIII1-5 was applied in PBS containing 0.05% tween and 0.1% BSA (PBST) (1 h, RT). TNCIII1-5 was detected with horseradish peroxidase (HRP)-anti-HIS (Abcam) in PBST (4 min, RT), developed with 3,3′,5,5′-tetramethylbenzidine (TMB) (eBioscience), stopped by sulfuric acid stop solution, and measured at 450 nm using a plate reader (A450) (Safire II, Tecan). Binding of TNCIII1-5 was analyzed with and without the presence of heparin (4 µM) (average MW of 15 kDa, Sigma). The plotted data is the A450 value of TNCIII1-5 binding subtracted by the A450 value in the absence of TNCIII1-5. An absorbance reading over 0.1 was considered as a significant interaction. To verify absorbance to the ELISA plate, a selection of growth factors that did not show binding to TNC III1–5 was detected with their respective antibodies.

### Competition assays

To analyze the binding of growth factors to TNCIII1–5 in the presence of heparin and the cross-competition between TNCIII1–5 and full length native human TNC (AbDSerotech) to bind growth factors, ELISA plates were coated with 50 nM growth factor (1 h, 37°C), blocked with 2% BSA (1 h, RT), and further incubated with 10 nM TNCIII1–5 in PBST in the presence of a gradient of heparin (0.01, 0.64, 3.2, 16, 80, 400, 2000, and 10000 nM) or full length TNC (0, 0.2, 0.6, 1.9, 5.6, 17, 50 nM) (1 h, RT). TNCIII1–5 was detected with HRP-anti-HIS in PBST (45 min, RT) and developed with TMB. Background values were obtained by applying heparin or TNC at the respective concentration in the absence of TNCIII1–5 and subtracted from the A450 value of TNCIII1–5 binding. The plotted data is the binding of TNCIII1–5 relative to TNCIII1–5 binding in the absence of heparin or TNC.

Binding of 10 nM TNC to growth factors of different families, namely PDGF-BB, BDNF, VEGF-A165, FGF-2 and BMP-2, was detected with mouse anti-TNC (1∶2,000, 45 min, RT) (Abcam, Cambridge, UK), a secondary HRP conjugated anti-mouse antibody (1∶2,000, 45 min, RT) (Dako), and TMB. TNC binding was analyzed and plotted after subtraction of the levels obtained without TNC.

### Surface plasmon resonance to quantify affinity values

Measurements were made using a Biacore X100 surface plasmon resonance instrument (GE Healthcare). TNCIII1–5 was immobilized in one channel using the amine coupling kit on a carboxylated gold chip (Sensor Chip C1; GE Healthcare), while BSA was similarly immobilized to the control channel. Approximately 450 RU of TNCIII1–5 was coupled to the chip according to the manufacturer's instruction. The reaction was quenched using 1 M ethanolamine. BSA was functionalized to the control channel at approximately 700 RU using the same method. After quenching of the reactive groups, PBS with 1% BSA was flowed over both channels to block any nonspecific interactions. Growth factors were diluted in the running buffer (PBS with 0.1% BSA) and delivered at a flow rate of 30 µl/min. PDGF-BB, FGF-2 and TGF-β were flowed at increasing concentrations from 0.4 nM to 33 nM, using 3-fold dilutions; NT-3 was flowed at increasing concentrations from 1.23 nM to 100 nM, using 3-fold dilutions. Binding constants of growth factors to TNCIII1–5 were automatically calculated using BIAevaluation software Biacore X100 (GE Healthcare) fitted using Langmuir binding kinetics.

### Identification of domains in TNCIII1–5 responsible for binding growth factors

A sandwich ELISA was performed to test the affinity of different domains present in TNCIII1–5 to PDGF-BB. The domains tested were GST-TNCIII3, GST-TNCIII4, GST-TNCIII5, GST-TNCIII3-4, GST-TNCIII4-5, and GST-TNCIII3-5. ELISA plates were coated with 50 µL of each domain (1 µM) in PBS, as well as GST only and BSA (1 h, 37°C). Wells were blocked using PBS with 2% BSA (1 h, RT) and then incubated with 2 nM PDGF-BB (1 h, RT). Binding of PDGF-BB was detected using an anti-PDGF-BB antibody (R&D, Minneapolis, MN) (1∶2,000, 45 min, RT), a secondary HRP conjugated anti-mouse antibody (1∶2,000, 45 min, RT), and TMB.

## Results

### Production of TNC fragments

The TNC fragment TNCIII1–5 engineered with a TG substrate domain and a HIS tag was produced by mammalian expression, resulting in a protein with a molecular weight of approximately 53 kDa (Figure 1, B). The amino acid sequence can be found in Figure 1C. The TG substrate domain allows immobilization within fibrin matrices if desired [21]. The LPS levels were below the detection limit (0.3 ng/ml).

The individual domains TNCIII3, TNCIII4, and TNCIII5, and various combinations, TNCIII3–4, TNCIII4–5, and TNCIII3–5, were expressed by bacterial expression as GST fusions and purified using a C-terminal HIS tag. GST was used as a fusion protein because it may stabilize the individual TNCIII domains (Figure 1, B).

Calculation of the isoelectric points of the different TNCIII domains revealed that most domains have a pI around 4, while TNCIII4 and TNCIII5 have a pI of 8.25 and 9.57, respectively (Figure 1, D). This correlates with their ability to bind highly negatively charged heparin, similarly to the FNIII12–14 domains of fibronectin [22], which also possess pIs between 9 and 10.

### Binding of TNCIII1–5 to growth factors

**Figure 2:**
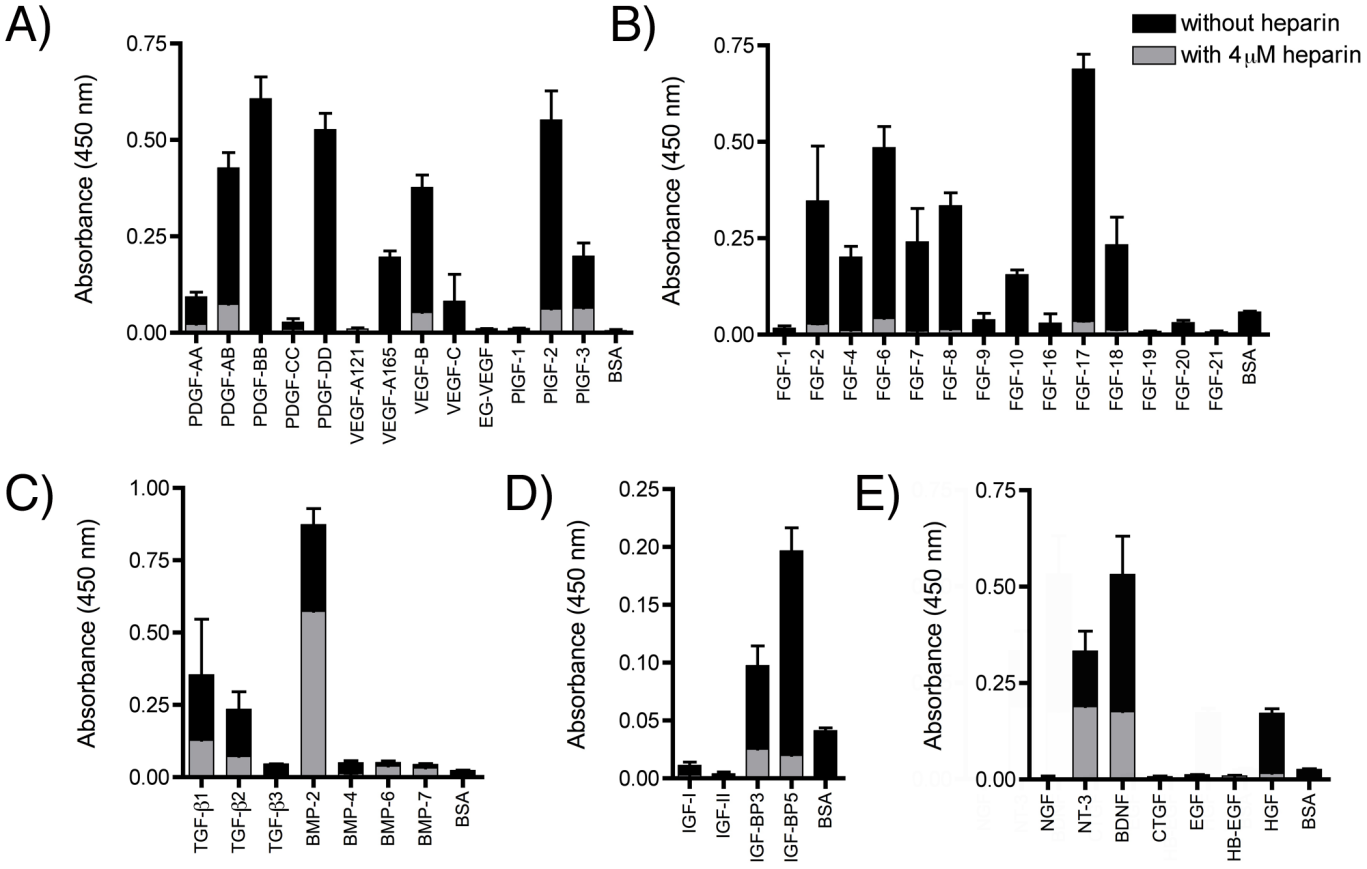
Identification of growth factors with an affinity towards TNCIII1–5. Binding of TNCIII1–5 to growth factors of different families, measured using indirect ELISA. Binding was measured in the presence of 4 µM heparin (grey) and in its absence (black). Various growth factor families were tested, PDGF/VEGF family (A), FGF family (B), TGF-β family (C), IGF family and IGF-BPs (D), and others (E) (n = 3, mean±SD).

An indirect ELISA method was used to screen binding of growth factors to TNCIII1–5 in a semi-quantitative manner, both in the absence of heparin and with excess (4 µM) heparin. In total, 43 growth factors plus two growth factor-binding proteins were screened, of which 23 growth factors and both growth factor-binding proteins were shown to bind (Figure 2). In general, excess heparin greatly reduced the binding interactions.

Many members from the PDGF/VEGF family, including PDGF-AA, PDGF-AB, PDGF-BB, PDGF-DD, VEGF-A165, VEGF-B, VEGF-C, PlGF-2, and PlGF-3 demonstrated binding to TNCIII1–5 (Figure 2, A). Whereas VEGF-A165 bound, its shorter splice variant VEGF-A121 did not, and whereas PlGF-2 and -3 bound, their shorter splice variant PlGF-1 did not.

From the FGF family, FGF-2, FGF-4, FGF-6, FGF-7, FGF-8, FGF-10, FGF-17, and FGF-18 demonstrated binding to TNCIII1-5 (Figure 2, B). Whereas FGF-2 (also known as basic fibroblast growth factor, bFGF) bound, FGF-1 (also known as acidic fibroblast growth factor, aFGF) did not.

Of the TGF-β family, TGF-β1 and TGF-β2, but not TGF-β3, bound to TNCIII1–5. Bone morphogenetic protein (BMP)-2 showed binding, but BMP-4, -6 or -7 did not bind (Figure 2, C).

From the insulin-like growth factor (IGF) family, although the growth factors IGF-1 and IGF-2 themselves did not bind, the high-affinity IGF-binding proteins IGF-BP3 and IGF-BP5 showed binding to TNCIII1–5 (Figure 2, D).

Of the neurotrophic factors, neurotrophin-3 (NT-3), brain-derived growth factor (BDNF), and nerve growth factor (NGF) were tested. Both NT-3 and BDNF were found to bind to TNCIII1–5, whereas NGF did not.

Furthermore, connective tissue growth factor (CTGF), epidermal growth factor (EGF), and heparin-binding EGF-like growth factor (HB-EGF) did not bind TNCIII1–5, but hepatocyte growth factor (HGF) did show affinity for TNCIII1–5 (Figure 2, E).

The effect of excess heparin on the binding of growth factors to TNCIII1–5 was also tested using the indirect ELISA, in the presence of a 40-fold excess (compared to TNCIII1–5) of heparin (4 µM) during incubation with TNCIII1–5 (100 nM). Binding of TNCIII1–5 to nearly all growth factors was greatly reduced in the presence of excess heparin. Only two growth factors retained more than 50% of their binding to TNCIII1–5, namely BMP-2 and NT-3, which retained 66% and 57% of there original binding, respectively (Figure 2). Growth factors of different families (e.g., VEGF-A121, PlGF-1, FGF-1, NGF) that did not bind TNCIII1–5 were verified to absorb to the ELISA plate using their respective antibodies and were easily detected, showing significant binding over background levels (Figure S1).

### Competition assay between heparin and growth factors to bind TNCIII1–5

**Figure 3:**
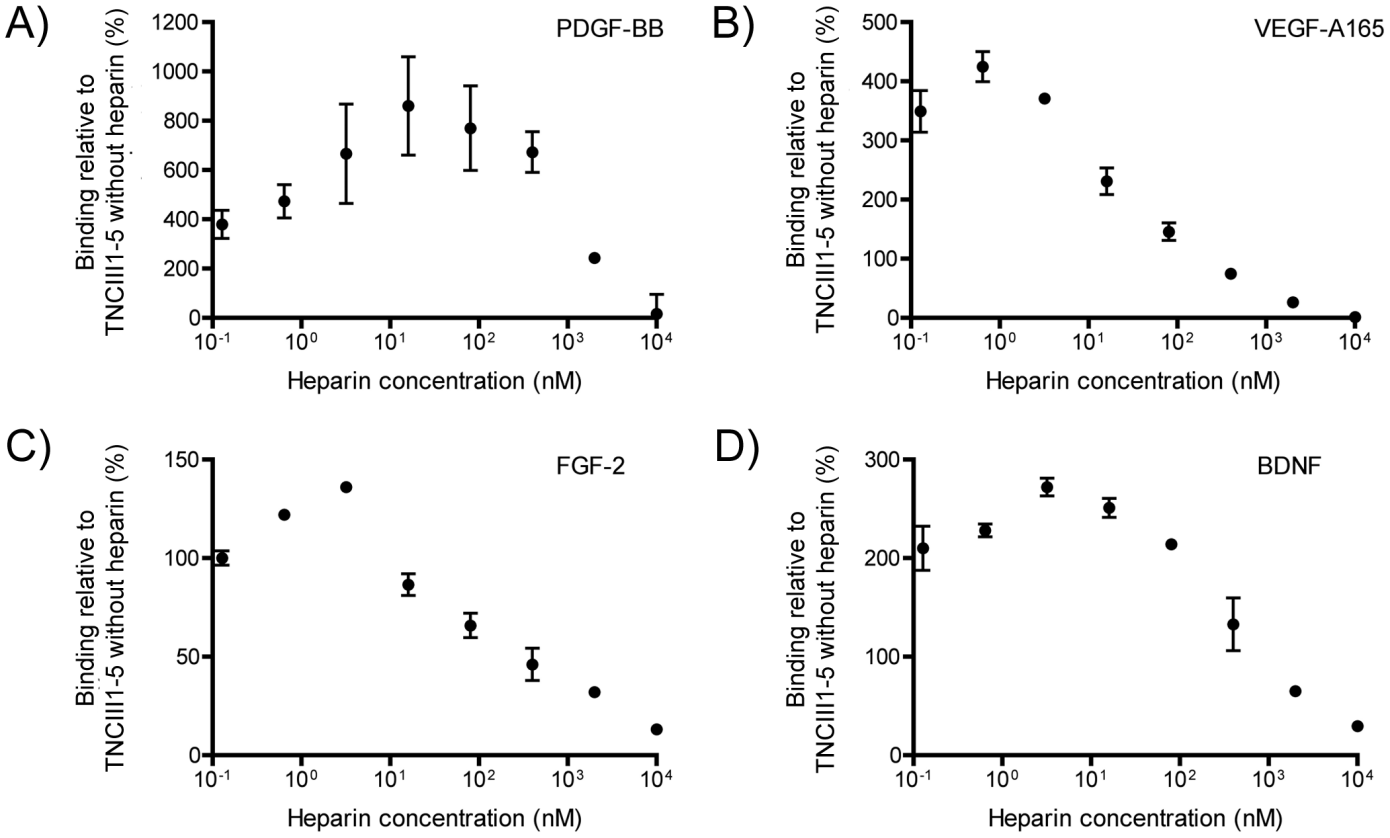
The effect of heparin concentration on the ability of TNCIII1–5 to bind selected growth factors. Using ELISA, the binding of TNCIII1–5 (10 nM) to various growth factors (A) PDGF-BB, (B) VEGF-A165, (C) FGF-2, and (D) BDNF was measured in the presence of increasing concentrations of heparin, 0.01 nM to 10,000 nM, with 100% representing binding of 10 nM TNCIII1–5 without the presence of heparin. The binding of the growth factors to TNCIII1–5 reached a maximum at an intermediate heparin concentration for all growth factors tested (n = 3, mean±SD).

To further investigate the influence of heparin on growth factor binding to TNCIII1–5, the indirect ELISA described above was used to measure binding at different concentrations of heparin. Selected growth factors of different families, namely PDGF-BB, VEGF-A165, FGF-2 and BDNF, were analyzed. A fixed amount of TNCIII1–5 (10 nM) was added with various dilutions of heparin (0.01 nM to 10,000 nM). At low concentrations of heparin, growth factor binding to TNCIII1–5 was enhanced by heparin addition, but with increasing concentrations of heparin, growth factor binding was diminished. The maximum binding of PDGF-BB and VEGF-A165 was in the presence of heparin at 16 nM and 0.64 nM, respectively (Figure 3, A–B), while maximum binding for FGF-2 and BDNF were observed at a heparin concentration of 3.2 nM (Figure 3, C–D); in all experiments, TNCIII1–5 was at 10 nM. High excesses of heparin, say 1000-fold (i.e., 10 µM), blocked essentially all growth factor binding to TNCIII1–5.

### Cross-competition assay between TNCIII1–5 and full length TNC

**Figure 4:**
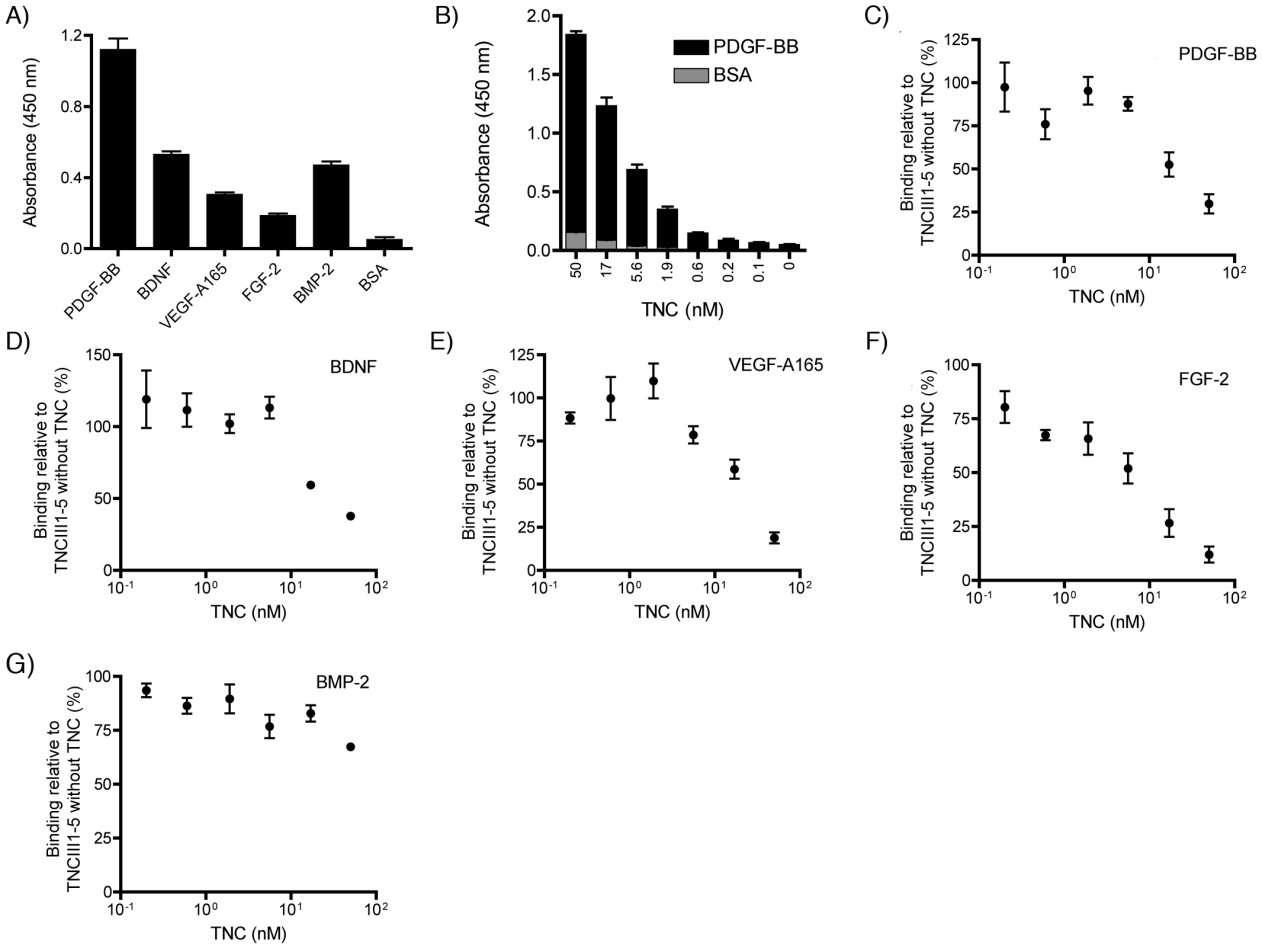
Full-length TNC and TNCIII1–5 cross-competition ELISA. Testing of binding of full length TNC to a selection of growth factors and cross-competition binding assay of TNC with TNCIII1–5. (A) Binding of TNC (10 nM) to PDGF-BB, BDNF, VEGF-A165, FGF-2, and BMP-2. (B) An increasing dose of full length TNC (0 to 50 nM) resulted in higher binding levels of TNC to PDGF-BB (black). No specific binding to BSA (grey) was observed. (C–G) Cross-competition assay between full length TNC and TNCIII1–5 (10 nM) to bind selected growth factors: PDGF-BB (C), BDNF (D), VEGF-A165 (E), FGF-2 (F), and BMP-2 (G). The TNC concentration varied from 0.1 nM to 50 nM (n = 3, mean±SD), with 100% representing binding of 10 nM TNCIII1–5 without the presence of TNC.

To determine if full-length TNC shares the same binding site as the TNC fragment TNCIII1–5, a cross-competition ELISA was performed with increasing concentrations of TNC (0 nM to 50 nM) at a fixed concentration of TNCIII1–5 (10 nM). First, binding of full-length TNC (10 nM) to growth factors of different families, namely PDGF-BB, BDNF, VEGF-A165, FGF-2 and BMP-2, was verified (Figure 4, A). Second, binding of different doses of TNC to PDGF-BB (with BSA as a control) was performed, and specific binding of the full-length protein was observed (Figure 4, B). In the cross-competition assay, TNC was found to reduce binding of TNCIII1–5 to PDGF-BB, BDNF, FGF-2 and VEGF-A165, but only marginally inhibited binding to BMP-2. Approximately 50% inhibition of TNCIII1–5 binding to PDGF-BB, BDNF, and VEGF-A165 was seen in the presence of 17 nM full-length TNC (Figure 4, C–E), while approximately 50% inhibition towards FGF-2 occurred at 5.6 nM of TNC (Figure 4, F). These values, close to 1∶1 with the 10 nM TNCIII1–5 used in the experiment, suggest that the binding of most of the growth factors to the fragment TNCIII1–5 properly represents binding to the native structure within full-length TNC. However, the lack of 50% inhibition even at 50 nM TNC (a 5-fold excess, at which 34% inhibition occurred) for BMP-2 suggests that in some instances, the interactions may be more complex (Figure 4, G).

### Surface plasmon resonance to quantify affinity values

Surface plasmon resonance (SPR) was used to determine the equilibrium binding constants (K_D_) for selected growth factors (TGF-β1, PDGF-BB, NT-3 and FGF-2) from four different families (Figure S2). The affinities for both TGF-β1 and NT-3 were approximately 20 nM (Table 1). For PDGF-BB and FGF-2, the sensorgrams did not fit Langmuir kinetics, displaying a non-standard kinetic profile.

**Table 1:** Equilibrium binding constants (K_D_), and association (k_on_) and dissociation (k_off_) rates were determined using surface plasmon resonance (SPR) and fitting the data to Langmuir binding kinetics for binding of immobilized TNCIII1–5 to TGF-β1 and NT-3.

Growth factor	k_on_ (1/Ms)	k_off_ (1/s)	K_D_ (nM)
TGF-β1	1.17E+04	2.37E−04	20.3
PDGF-BB	NA*	NA*	NA*
NT-3	5.07E+04	1.06E−03	21.0
FGF-2	NA*	NA*	NA*

* Not applicable (NA) because the data was not interpretable using standard Langmuir binding kinetics for FGF-2 and PDGF-BB.

### Identification of domains within TNCIII1-5 responsible for binding growth factors

**Figure 5:**
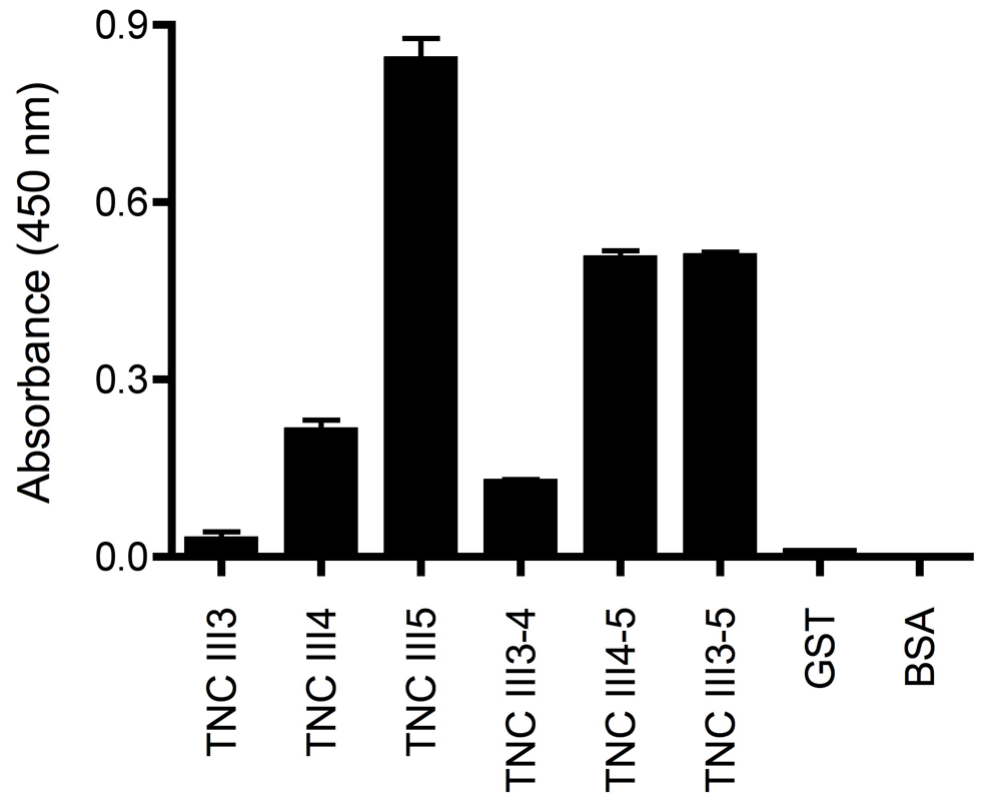
Determination of the specific growth factor-binding domain. The binding affinity of different (fusion) domains (GST-TNCIII3, GST-TNCIII4, GST-TNCIII5, GST-TNCIII3-4, GST-TNCIII4-5, GST-TNCIII3-5) to PDBF-BB was determined and compared to controls, GST only and BSA, using an indirect ELISA method. The TNC domain responsible for growth factor binding was mainly identified as domain TNCIII5, with TNCIII4 contributing to a lesser extent. (n = 3, mean±SD).

Individual domains of TNC (TNCIII3, TNCIII4, TNCIII5) and various combinations (TNCIII3–4, TNCIII4–5, TNCIII3–5) were expressed as GST fusions to identify which domains are responsible for growth factor binding, using PDGF-BB as an exemplary growth factor (Figure 5). The TNCIII3 showed no binding to PDGF-BB. TNCIII4 showed binding to PDGF-BB at a modest level, as did TNCIII3–4. By contrast, TNCIII5 showed high binding levels, 4-fold that of TNCIII4. Other combinations containing TNCIII5, namely TNCIII4–5 and TNCIII3–5, also showed high binding levels. When the GST fusion of these proteins was removed, the binding was significantly reduced (data not shown), which led us to hypothesize that the GST fusion may stabilize the folding of the TNCIII domains in a similar manner as observed with other fusion protein that stabilize the protein partner [23], [24]. Therefore, we produced TNCIII1–5 in order to generate a protein comprised of only domains from TNC and still maintain binding to growth factors, independent of a GST stabilizing domain.

Based on these results, we may conclude that within the TNCIII1–5 growth factor-binding fragment, binding is principally localized in the TNCIII5 domain.

## Discussion

The extracellular microenvironment is composed of numerous matrix-forming proteins that serve multiple functions, such as structural control and integrin binding for cellular stability. An increasingly observed role of ECM proteins is the ability to bind and sequester growth factors, acting as a reservoir for the stimulatory molecules that can be released during proteolytic remodeling of wounded areas [25], [26]. Additionally, an emerging concept for the role of growth factor binding within the ECM is the enhancement of cellular signaling through co-association of integrins and growth factor receptors [27].

In a recent report from our group, we showed that the FNIII12–14 domain of fibronectin possessed a highly promiscuous growth factor-binding ability [19]. FNIII12–14 was initially established as a heparin-binding domain [22], and later it was observed that the domain also binds VEGF-A165 [28]. Interestingly, TNCIII4–5 is also a known heparin-binding domain [17], [18], so we hypothesized that this domain may also bind a wide-range of growth factors. Human TNCIII1–5, indeed, demonstrated strong binding to many growth factors from different families; binding was located principally to TNCIII5, but TNCIII4 also contributed to the interaction. The cross-competition assay between TNCIII1–5 and full-length TNC confirmed that TNC can specifically block the interaction of the TNC subdomain with growth factors.

Even though FNIII12–14 and TNCIII1–5 are both, respectively, fibronectin type III and fibronectin type III-like repeats that bind heparin and interact with many of the same growth factors, there are also many growth factors that interact with only one of the domains. FNIII12–14 has been shown to bind to PDGF-CC, BMP-7, FGF-9, and CTGF, while TNCIII1–5 did not have a significant interaction with any of these growth factors. Conversely, TGF-β2 was found to bind to TNCIII1–5, which was reported to not interact with FNIII12–14 [19]. The biological significance of these differences is not known but could play a role in patterning of growth factors within the ECM, thus creating unique microenvironments for various cell types.

Additionally, an observed difference between growth factors that bound to TNCIII1–5 and those that did not was the presence of a heparin-binding domain within the growth factors. For example, VEGF-165 and PlGF-2, which contain a heparin-binding domain, were found to bind to TNCIII1–5, while VEGF-121 and PlGF-1, which lack a heparin-binding domain, did not. Because the heparin binding domains on both the growth factors and TNCIII1–5 are positively charged, the interactions cannot be attributed to simple charge-charge interactions. However, not all heparin binding growth factors interacted with TNCIII1–5, for example, FGF-1 and HB-EGF.

SPR was used to analyze the kinetics of binding of four selected growth factors from different families, namely PDGF-BB, NT-3, TGF-β1, and FGF-2. Both TGF-β1 and NT-3 displayed equilibrium dissociation constants of approximately 20 nM. The binding parameters of PDGF-BB and FGF-2 could not be determined using Langmuir kinetics because of the non-standard kinetic profile of the sensorgram curves. The kinetic profile could be the result of a complex binding interaction of the growth factors to the TNCIII domains, which may be due to different conformational changes induced during the binding, similar to those observed upon FNIII binding interactions of fibronectin [29]. The reported affinities of the examined growth factors towards FNIII12–14 [19] were slightly higher than those measured for TNCIII1–5, but generally within the same order of magnitude.

Interestingly, heparin plays a complex role in the interaction of growth factors to TNCIII1–5. At approximately equimolar concentrations of heparin and TNCIII1–5, the binding interaction was enhanced. For PDGF-BB and VEGF-A165, this effect increased binding by more than 2-fold. At greater concentrations of heparin, growth factor binding was reduced and was almost negligible at the maximum concentration of 10 µM heparin. Heparin has been reported to induce conformational changes within the heparin-binding domain of fibronectin, resulting in enhanced binding of VEGF-A165 [29] and PDGF-AA [30]. In addition, FNIII12–14 demonstrated enhanced binding for almost all binding growth factors with an excess of heparin [19]. Heparin may play a similar role in the binding interaction between TNCIII1–5 and growth factors, although in the case TNCIII1–5, heparin was found to significantly inhibit the binding at higher concentrations, in contrast to the behavior observed for FNIII12–14 [19].

The close proximity of an integrin-binding domain and a growth factor-binding domain within fibronectin causes the cooperative cellular signaling in response to fibronectin-binding growth factors [27]. A synergistic interaction has also been observed for VEGF-A165 [31], PDGF-AA [30], and PDGF-BB [32] with fibronectin, and for PDGF-BB with TNC [33]. Similarly to fibronectin, the growth factor-binding domain of TNC we identified is located proximal to an important integrin-binding domain, TNCIII3, which is known to bind integrins such as α_8_β_1_ and α_v_β_3_ [3]. These proximal interactions may explain the observation that TNC enhances PDGF-BB-induced proliferation and migration of smooth muscle cells by promoting crosstalk signaling between the PDGF-BB receptor (PDGF Rβ) and integrin α_v_β_3_ [33].

Even though the complete gene knockout of TNC has minimal phenotype in mice [34], [35], more recent studies revealed several defects in TNC knockout mice in the case of pathological intervention [36]. Growth factor binding to extracellular matrix components is a highly redundant phenomenon; for example fibronectin and collagen have been demonstrated to show relevant binding to growth factors [19], [37]. Therefore, we believe that the finding that tenascin binds growth factors may play an important role in multiple physiological and pathological processes that were not necessarily observed in the TNC knockout animals, but where tenascin does play a role. TNC plays an active role in development, nerve growth, cancer, and wound healing, and thus may be involved in the regulation and signaling of growth factors to control cell proliferation and differentiation, as well as signaling directly through its multiple integrin binding domains. For example, S100A4(+) fibroblasts have been reported to play a role in metastatic colonization due to their production of both VEGF-A165 and TNC [14]. Based on the interactions identified herein, there may be an important relationship between TNC and VEGF-A165 affecting metastatic colonization. Additionally, it has recently been reported that lower levels of TGF-β1 and reduced epithelial-mesenchymal transition (EMT) were observed after eye lens injury in TNC^−/−^ mice [38]. This observation may be related to the ability of TNC to bind to TGF-β1, which could locally sequester the growth factor at the site of injury.

TNC has been shown to play a broad role in pathophysiology [11], and our observation of a promiscuous interaction with growth factors suggests that molecular interactions between TNC and growth factors may be important in this role. Recently, it has been shown that breast cancer cells producing TNC promote the survival and outgrowth of pulmonary micrometastases by enhancing the expression of stem cell signaling components [15]. It has been suggested that TNC may promote cell dissemination and survival during early tumor metastasis by sequestering growth factors, ECM molecules, enzymes, cell surface receptors and integrins [39], even though specific binding of growth factors to TNC had not yet been reported. The identification of the TNC growth factor-binding domain opens new opportunities to investigate its role in disease.

In addition, the promiscuous binding of growth factors to TNC could play an important role during development and tissue repair when TNC is upregulated at the site of injury. The production and use of specific functional TNC domains may therefore be attractive in the field of tissue engineering to create biomimetic materials [26], [40]. Scaffolds could be modified with these engineered TNC fragments to function as cell signaling platforms or drug delivery devices by retaining and presenting growth factors within the matrix [41], [42], as our group has done with functional FN fragments [27].

## Conclusion

In this report, we found that TNC binds growth factors via its fourth and principally fifth fibronectin type III-like domain with nanomolar affinity. TNCIII1–5 was produced and shown to bind a wide variety of growth factors from different families, including the PDGF family, the FGF family, the TGF-β superfamily, and neurotrophins, in addition to the IGF-BPs. This promiscuous affinity for growth factors from diverse families may be related to the broad role played by TNC in both tissue repair and disease.

## Supporting Information

Figure S1 **Growth factor detection on ELISA plate.** Growth factors of different families (e.g., VEGF-A121, PlGF-1, FGF-1, NGF) that did not bind TNCIII1-5 were verified to absorb to the ELISA plate, showing significant binding over background levels.
(TIF)

Figure S2 **Affinity measurement of TNCIII1-5 to representatives from four growth factors families.** Surface plasmon resonance measurements to quantify affinity values were made using a Biacore X100 surface plasmon resonance instrument. TNCIII1-5 was immobilized in one channel, while BSA was similarly immobilized to the control channel. Growth factors were diluted in the running buffer (PBS with 0.1% BSA) and delivered at a flow rate of 30 µl/min. (A) TGF-β1, (B) PDGF-BB and (D) FGF-2 and were flowed at increasing concentrations from 0.4 nM to 33 nM, using 3-fold dilutions; (C) NT-3 was flowed at increasing concentrations from 1.23 nM to 100 nM, using 3-fold dilutions. Binding constants of growth factors to TNCIII1-5 were automatically calculated using BIAevaluation software Biacore X100 (GE Healthcare) fitted using Langmuir binding kinetics.
(TIF)
