# Supplementary figures and images for: Tenascin C Promiscuously Binds Growth Factors via Its Fifth Fibronectin Type III-Like Domain
Source: PLoS One. 2013 Apr 18;8(4):e62076. doi: 10.1371/journal.pone.0062076 (PMC3630135; doi:10.1371/journal.pone.0062076)

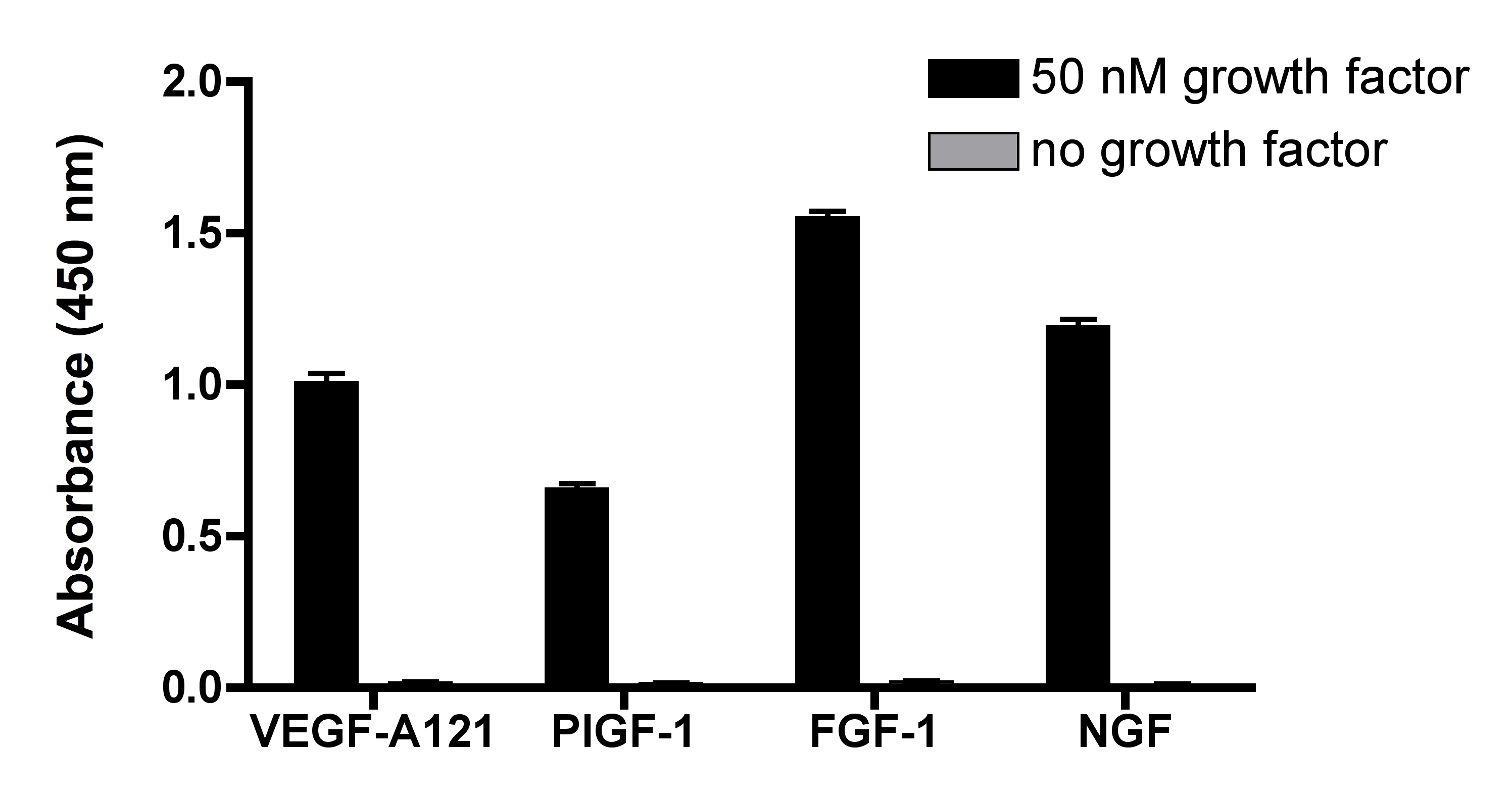

Supplement: Figure S1 — Growth factor detection on ELISA plate. Growth factors of different families (e.g., VEGF-A121, PlGF-1, FGF-1, NGF) that did not bind TNCIII1-5 were verified to absorb to the ELISA plate, showing significant binding over background levels. (TIF) [file pone.0062076.s001.tif]

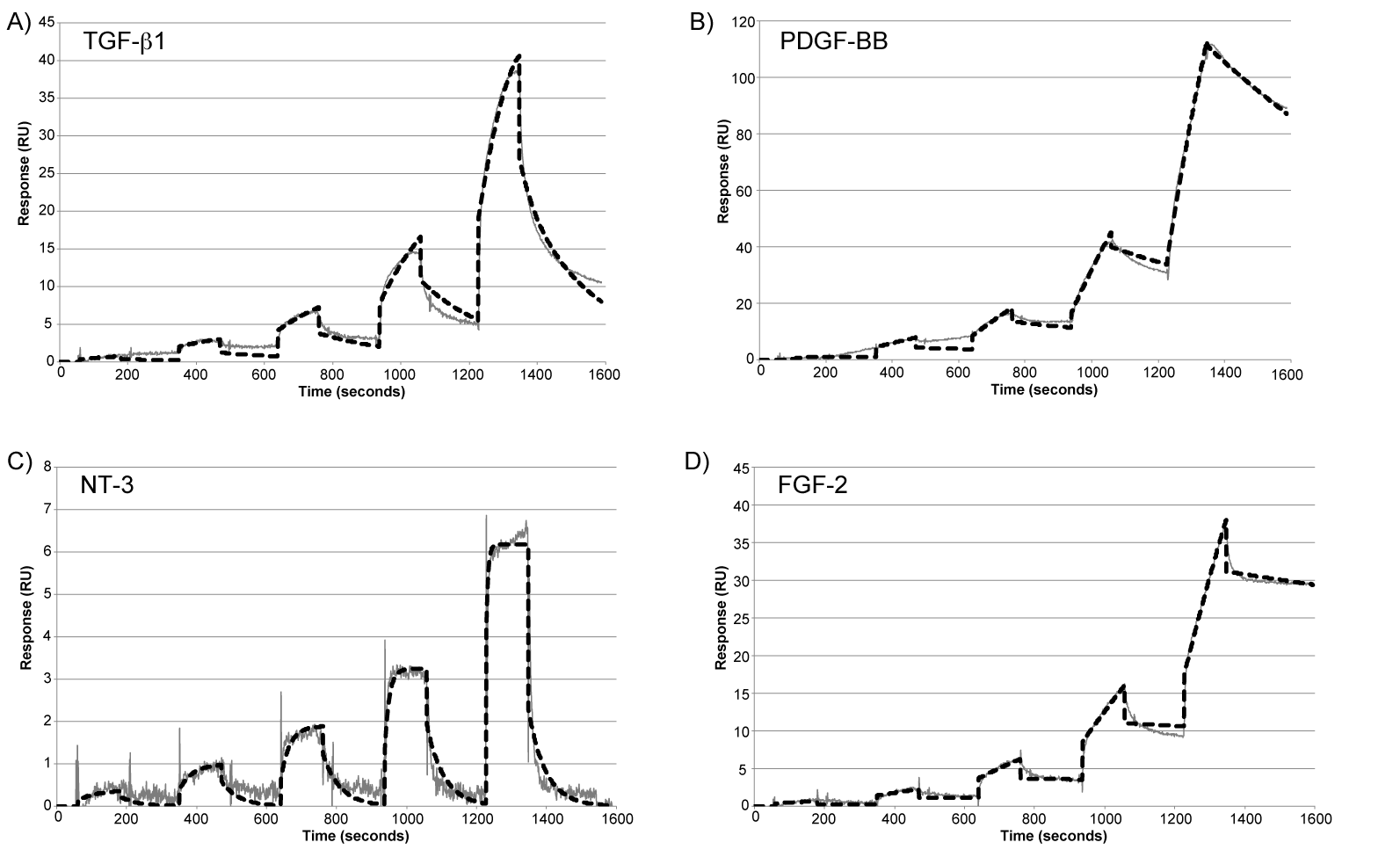

Supplement: Figure S2 — Affinity measurement of TNCIII1-5 to representatives from four growth factors families. Surface plasmon resonance measurements to quantify affinity values were made using a Biacore X100 surface plasmon resonance instrument. TNCIII1-5 was immobilized in one channel, while BSA was similarly immobilized to the control channel. Growth factors were diluted in the running buffer (PBS with 0.1% BSA) and delivered at a flow rate of 30 µl/min. (A) TGF-β1, (B) PDGF-BB and (D) FGF-2 and were flowed at increasing concentrations from 0.4 nM to 33 nM, using 3-fold dilutions; (C) NT-3 was flowed at increasing concentrations from 1.23 nM to 100 nM, using 3-fold dilutions. Binding constants of growth factors to TNCIII1-5 were automatically calculated using BIAevaluation software Biacore X100 (GE Healthcare) fitted using Langmuir binding kinetics. (TIF) [file pone.0062076.s002.tif]
